# Simultaneous removal of fluoride and arsenic in geothermal water in Tibet using modified yak dung biochar as an adsorbent

**DOI:** 10.1098/rsos.181266

**Published:** 2018-11-21

**Authors:** Luo Chunhui, Tian Jin, Zhu Puli, Zhou Bin, Bu Duo, Lu Xuebin

**Affiliations:** 1 School of Environmental Science and Engineering, Tianjin University, Tianjin 300072, People's Republic of China; 2 The Administrative Center for China's Agenda21, Beijing 100038, People's Republic of China; 3 Department of Chemistry and Environmental Science, School of Science, Tibet University, Lhasa 850000, People's Republic of China

**Keywords:** fluoride, arsenic, geothermal water, yak dung biochar, Tibet

## Abstract

Fluoride (F) and arsenic (As) are two typical and harmful elements that are found in high concentrations in geothermal water in Tibet. In this work, yak dung, an abundant source of biomass energy in Tibet, was made into biochars (BC1, BC2 and BC3) by pyrolysis under different conditions, and the better biochar was modified by FeCl_2_ (Fe-BC3). The adsorption conditions were optimized to adsorb F and As in geothermal water. The results showed that BC3 can remove 90% F^−^ and 20% As(V), which is the best effect of the three initial biochars. Fe-BC3 could remove 94% F^−^ and 99.45% As(V) under the same conditions as BC3, which was an adsorbent dosage 10 g l^−1^, pH 5–6 and temperature of 25°C. It was also demonstrated that the removal rate did not decrease at 80°C. A quasi-second-order kinetic model best described the adsorption behaviour of ions on the surface of the biochar. The maximum adsorption capacity of F^−^ and As(V) on Fe-BC3 was 3.928 mg g^−1^ and 2.926 mg g^−1^, respectively. The features of Fe-BC3 were characterized by X-ray diffraction, Fourier transform infrared, Brunauer–Emmett–Teller, energy-dispersive spectrometer and scanning electron microscopy to understand the adsorption process.

## 1. Introduction

The amount of geothermal resource reserves in Tibet is the highest in China. The potential of geothermal resources for power generation exceeds 1 million kilowatts [1]. In addition to power generation, geothermal resources have a wide range of applications in heating houses, vegetable greenhouses, medical treatment and bathing. When geothermal water is generated or used, it is either recharged or discharged directly to surface waters. The recharge causes corrosion and clogging of the pipes. If geothermal water drains directly into adjacent rivers after it is discharged from the surface, it seriously affects the water quality of the river. In addition, local residents use and even drink untreated geothermal water and river water. Fluoride (F) and arsenic (As) are two typical and harmful elements that are high in concentration in geothermal water. The concentration of F in geothermal waters in Yangbajing, a typical high-temperature geothermal area in China, can be as high as 19.6 mg l^−1^, which is ten times the World Health Organization (WHO) regulation. The concentration of As can be as high as 3.56 mg l^−1^, which is more than 350 times the upper limit for drinking water. The WHO provides an upper limit of 1.5 mg l^−1^ for F concentration in drinking water and an upper limit of 0.01 mg l^−1^ for As concentration [2]. The content of harmful elements in geothermal water is sufficiently high that if people directly drink the surface water or groundwater affected by geothermal water, their health may be affected to varying degrees.

In recent years, scholars worldwide have performed a substantial amount of research on the removal of fluoride and arsenic in wastewater. At present, the most mature technologies are as follows: precipitation, adsorption, ion exchange and membrane technology. The adsorption method is a simple and easy water treatment technology and is the most widely used in China. At present, adsorbents for adsorbing fluoride and arsenic that are commonly used in water treatments are activated alumina, bone charcoal and zeolite [3]. Although activated alumina has a good removal effect for fluoride and arsenic, the pH value must be adjusted during the treatment, which restricts its application. The adsorption capacity of bone charcoal and natural zeolite on fluoride and arsenic is also quite limited. Therefore, the development of many low-cost and effective adsorbents is an important research topic so that a solution for water pollution can be found using the adsorption method. As a new type of adsorbent, biochar is characterized by high porosity, high stability, large specific surface area, strong adsorption capacity and the availability of many sources of raw materials. Therefore, researchers have paid increasingly more attention to the development and utilization of biochars in recent years. Common biochars are mainly produced from rice husk carbon, straw charcoal, bamboo charcoal, animal faeces charcoal and so on. At present, biochar and its composite materials have been widely used in the adsorption of inorganic pollutants (heavy metals, fluorine, etc.) and organic pollutants in the environment [4].

Yu Zhihong *et al.* [5] used biochar-manganese oxide composites made from corn stalk biochar and potassium permanganate solution to adsorb As(III) in water, resulting in a maximum adsorption capacity ranging from 11.41 to 20.08 mg g^−1^. Zhang Feng *et al.* [6] used iron-loaded biochar to effectively adsorb As(V) in the water. Wang Shengsen *et al.* [7] synthesized magnetic biochar by pyrolysing a mixture of natural, hematite minerals and pine biomass. Compared with unmodified biochar, hematite-modified biochar not only had stronger magnetism but also showed a greater ability to remove As in aqueous solution. Lin Lina *et al.* [8] impregnated the original biochar with Fe–Mn oxide to increase the specific surface area of biochar, and the interaction of manganese oxide and oxygen-containing functional groups on biochar promoted the conversion of As(III) to As(V). Evita Agrafioti *et al.* [9] modified biochar with Ca and Fe to effectively remove As(V) from aqueous solutions.

In Tibet, yak dung provides an abundant source of biomass energy: the annual output is approximately 7.73 million tons [10]. Local residents burn yak dung directly for fuel, causing serious air pollution. The purpose of this study was to use the abundant yak dung as a raw material to make biochars by pyrolysis for the removal of fluoride and arsenic from geothermal water. This research provides a way to solve the problem of fluoride and arsenic pollution in geothermal water by using local resources in Tibet.

## 2. Material and methods

### 2.1. Experimental materials

Yak dung was obtained from a pasture in the plateau area of Eastern Tibet. The dung was air-dried, crushed to sieve through a #60 mesh and dried in an oven at 80°C for 2 h for subsequent experiments. Fluorine standard solution (1000 mg l^−1^) was purchased from Tianjin Kwangfu Fine Chemical Industry Research Institute, and arsenic standard solution (1000 mg l^−1^) was purchased from Shanghai ANPEL Experimental Polytron Technologies Inc.

### 2.2. Biochar preparation from yak dung

A ceramic ark was filled with yak dung and placed in a vacuum tube furnace (Tianjin Mafuer Technology Co. Ltd., TL1200). Pyrolysis was conducted under a nitrogen atmosphere in order to maintain anaerobic conditions. The pyrolysis temperature was set to 500°C, with a rate of temperature increase of 200°C h^−1^, 400°C h^−1^, or 600°C h^−1^, respectively; after reaching the target temperature, the sample was kept in the tube furnace for 60 min, 120 min, or 180 min, respectively. Afterwards, the biochars were removed from the furnace when cooled to room temperature. The raw biochars were washed three times with 1 mol l^−1^ HNO_3_ (the solid-to-liquid ratio was 1 : 25) for 10 min and then washed with ultrapure water until the filtrate pH became neutral [11]. The resulting solid was dried at 80°C and stored in a desiccator. The biochar prepared at 500°C at 200°C h^−1^ for 60 min was labelled BC1, the biochar prepared at 500°C at 400°C h^−1^ for 120 min was labelled BC2, and the biochar prepared at 500°C at 600°C h^−1^ for 180 min was labelled BC3.

BC3 was modified with FeCl_2_ using the following process [12]: BC3 was added into 0.1 mol l^−1^ FeCl_2_ with a solid-to-liquid ratio of 1:15 and then stirred with a magnetic stirrer for 24 h; NaClO solution was added every 6 h at a mass-to-volume ratio of 1 g FeCl_2_·4H_2_O to 2 ml NaClO; 1 mol l^−1^ HCl or NaOH was used to adjust the pH of the solution to maintain a value between 4.5 and 5.0; and finally, the obtained solid, which was labelled Fe-BC3, was washed with ultrapure water and dried until use.

### 2.3. Adsorption and analysis method

Investigations into As (approx. 3.6 mg l^−1^) and F^−^ (approx. 19.0 mg l^−1^) sorption kinetics with biochar were performed as follows. Briefly, approximately 0.30 g of biochar was added to 30 ml of sorbate solution in a 50 ml plastic container at 25°C. Thus, the amount of adsorbent added for all treatments was 10 g l^−1^. The vessels were placed into a constant temperature, oscillating water bath and shaken at 150 rpm until sampling. At each sampling time (0, 5, 10, 15, 20, 30, 40, 50, 60, 120, 240, 360, 480, 600 and 720 min), the suspensions were immediately filtered through a nylon membrane filter with a pore size of 0.45 µm. The values of all samples were measured in triplicate and averaged.

The As in the filtrates was determined with an inductively coupled plasma-optical emission spectrometer (ICP-OES, Thermo, 7000 series), and F^−^ was measured with a fluoride ion meter (Rex Electric Chemical, PXSJ-216). Adsorbed As and F^−^ were calculated as the difference in concentration between the initial and final solution.

### 2.4. Kinetics and isotherm analysis

#### 2.4.1. Kinetic

Based on the experimental results, the adsorption kinetics of As(V) and F^−^ onto BC3 and Fe-BC3 were fitted using two kinetic models: a quasi-first-order model and a pseudo-second-order model.

The quasi-first-order kinetic equation is as follows [13]:

2.1 $$\ln(q_{e}-q_{t})\;=\;\ln q_{e}-k_{1}t,$$

where *q_t_* (mg g^−1^) is the amount adsorbed per unit mass at time *t* (*h*), *q_e_* (mg g^−1^) is the theoretical value for adsorption capacity and *k*_1_ (*h*^−1^) is the quasi-first-order adsorption rate constant. The values of *q_e_* and *k*_1_ can be obtained from the slope and intercept of the linear plot of ln (*q_e_* − *q_t_*) versus *t*.

The pseudo-second-order kinetic equation is as follows [14]:

2.2 $$\frac{t}{q_{t}}=\frac{1}{k_{2}q_{e}^{2}}+\frac{t}{q_{e}},$$

where *k*_2_ (g mg^−1^ h^−1^) is the rate constant of the pseudo-second-order model. The values of *q_e_* and *k*_2_ can be obtained from the intercept and slope of the linear plot of *t*/*q_t_* versus *t*.

#### 2.4.2. Isotherm

Adsorption isotherms were constructed using 30 ml solutions in which As(V) and F^−^ concentrations ranged from 5 to 40 mg l^−1^ with a biochar concentration of 10 g l^−1^. The suspensions were shaken on a shaker for 24 h and then treated as described above. Isotherm data were simulated with various isotherm models.

In this study, Freundlich and Langmuir models were used to fit the experimental data. The Freundlich isotherm equation (equation (2.3)) assumes that the surface of the adsorbent is heterogeneous, that adsorption is multilayered and that adsorption capacity gradually decreases as adsorption sites are filled [14].

2.3 $$\mathrm{lg}q_{e}=\;\frac{1}{n}\mathrm{lg}C_{e}+\;\mathrm{lg}K_{F},$$

where *C_e_* is the equilibrium concentration of ions (mg l^−1^), *q_e_* is the equilibrium adsorption capacity (mg g^−1^), *K*_F_ is the Freundlich constant denoting adsorption capacity (mg g^−1^) and *n* is an empirical constant that represents the adsorption intensity. The Freundlich constants *K*_F_ and *n* can be calculated from the intercept and slope of the linear plot of log *q_e_* versus log *C_e_*.

The Langmuir isotherm equation (equation (2.4)) assumes that the surface of the adsorbent is homogeneous and that adsorption occurs as a monolayer. The interaction between the adsorbed particles is negligible, and the adsorption energies are uniform across all sorption sites [15].

2.4 $$\frac{C_{e}}{q_{e}}=\frac{C_{e}}{q_{m}}+\frac{1}{K_{L}q_{m}},$$

where *q_m_* is the maximum adsorption capacity (mg g^−1^), and *K_L_* is the Langmuir constant (mg g^−1^) and is related to the energy of adsorption. *C_e_* and *q_e_* are the same as above. The maximum adsorption capacity *q_m_* and *K_L_* can be calculated from the slope and intercept of the linear plot of *C_e_*/*q_e_* versus *C_e_*.

### 2.5. Biochar characterization analysis

Total surface area was measured using N_2_ sorption on a NOVA 1200 analyser and calculated using the Brunauer–Emmett–Teller (BET) method. Scanning electron microscopy (SEM) images were obtained with a scanning electron microscope (Hitachi, S-4800). Energy dispersive X-ray spectroscopy (EDS) (Hitachi) was coupled with SEM to examine the surface elemental composition and obtain surface elemental distribution maps. Surface crystallinity was analysed to identify Fe-bearing minerals using an X-ray diffractometer (XRD) (Japanese Science Company, D/MAX-2500). Functional groups were measured using Fourier transform infrared (FTIR) spectroscopy (Shimadzu Corporation, IRAffinity-1S).

## 3. Results and discussion

### 3.1. Effects of pH and temperature on F and As removal rate by different biochars

**Figure 1.**
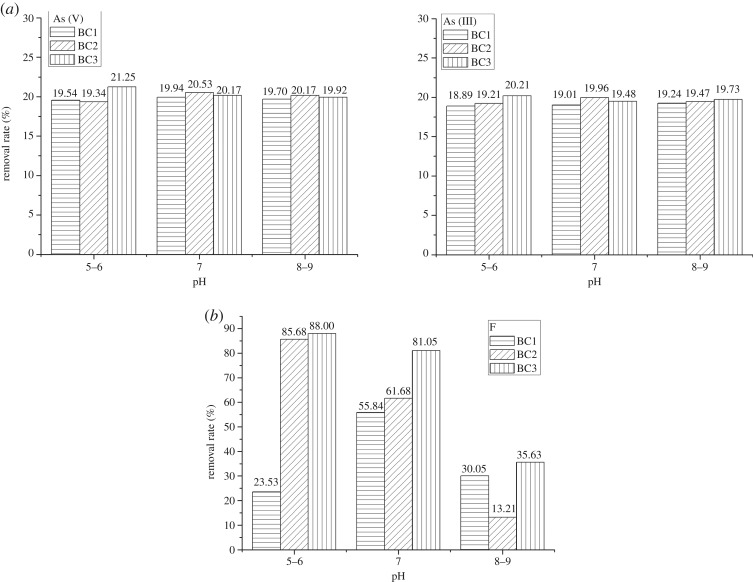
Removal rates of (*a*) As (V) and As (III) and (*b*) F under different pH (5–6, 7, 8–9) at 25°C by BC1, BC2 and BC3.

BC1, BC2 and BC3 were added into a solution of As(V), As(III) and F^−^ at 25°C and at different pH values (5.0–6.0, 7.0, 8.0–9.0) to complete the adsorption experiment. Figure 1 illustrates the removal rates of As(V), As(III) and F^−^. The removal rates of As(V) and As(III) for the three biochars under different pH values (5.0–6.0, 7.0, 8.0–9.0) were similar and relatively low, approximately 20% (figure 1*a*). Considering that the existence of most arsenic in nature is arsenate, As(V) was the final research object in this work. The removal rate of F^−^ (figure 1*b*) was the highest (approximately 88.0%) for BC3 at pH = 5–6, whereas the removal rate of the other two biochars was comparatively poor. It can be concluded that BC3 was the most effective of the biochars at adsorption at pH = 5.0–6.0. Table 3 shows the surface area, average pore size and total pore volume of BC1, BC2, BC3 and Fe-BC3. The specific surface area of BC3 is the largest at 100.316 m^2^ g^−1^. The specific surface areas of BC1 and BC2 are only 37.944 m^2^ g^−1^ and 49.687 m^2^ g^−1^, respectively, which reveals why these biochars are not as effective at adsorption as BC3. In addition, at acidic pH values, surface functional groups such as amino, carboxyl and thiol impart a positive charge on the surface due to protonation. Asheesh *et al*. [16] studied the effects of bagasse, wood sawdust and wheat straw biochar on fluoride, and the best pH was approximately 6.0.

**Figure 2.**
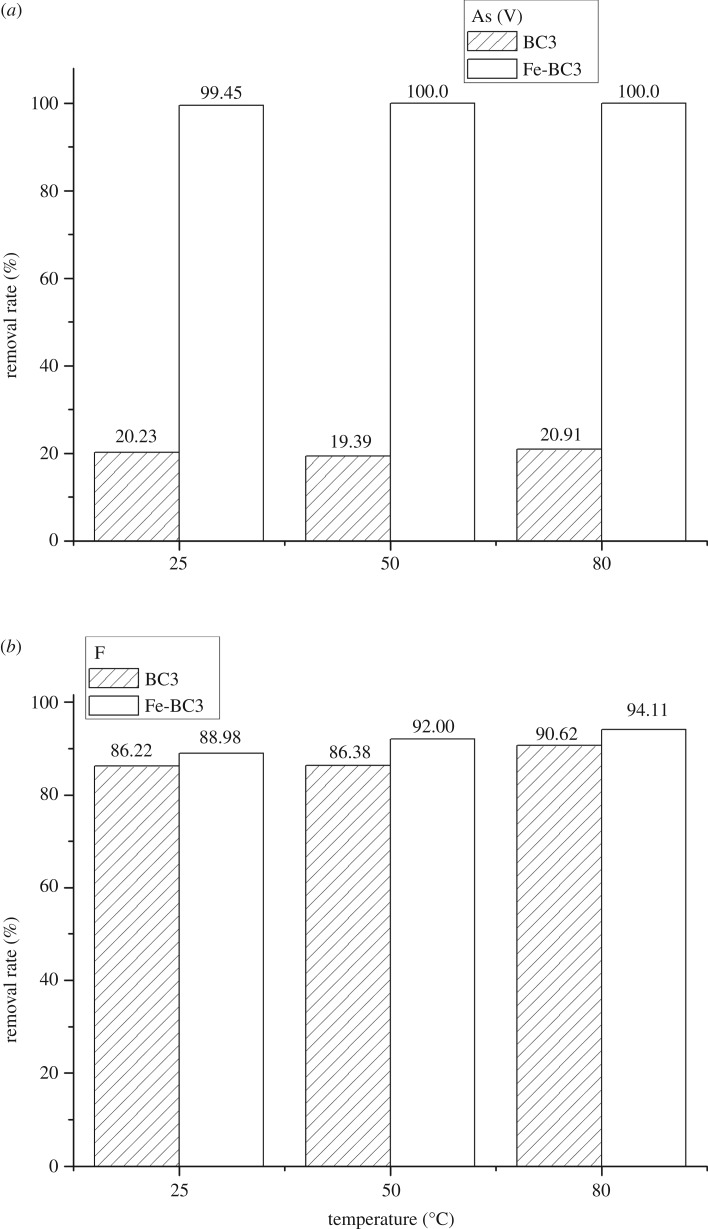
Removal rates of (*a*) As (V) and (*b*) F^−^ at different temperatures (25°C, 50°C, 80°C) under pH = 5–6 by BC3 and Fe-BC3.

BC3 and Fe-BC3 were added into As(V) and F^−^ solutions at different temperatures (25, 50, 80°C) and with pH = 5.0–6.0. Figure 2 illustrates the removal rates of As(V) and F^−^ at different temperatures. The results showed that the temperature of the solution had a minimal effect on the removal of these elements. This observation demonstrates that geothermal water can be treated directly by the appropriate biochars without requiring cooling. The removal rates of F^−^ for Fe-BC3 increased slightly compared to that for BC3, approximately 4.0% (figure 2*b*), while the removal rates of As(V) increased greatly, approximately 80.0% (figure 2*a*). Figure 9 shows the Energy Dispersive Spectrometer (EDS) analysis results for BC3 and Fe-BC3. The EDS results indicate that the content of Fe increases and that Cl appears on Fe-BC3, which illustrates that Fe and Cl were incorporated into BC3 when it was impregnated with FeCl_2_. The results also indicate that Fe plays a pivotal role in the adsorption of As(V), which is probably because the presence of Fe increases the complexation and electrostatic interaction between As(V) and biochar [17].

### 3.2. Adsorption kinetics and isotherm

#### 3.2.1. Adsorption kinetics

**Figure 3.**
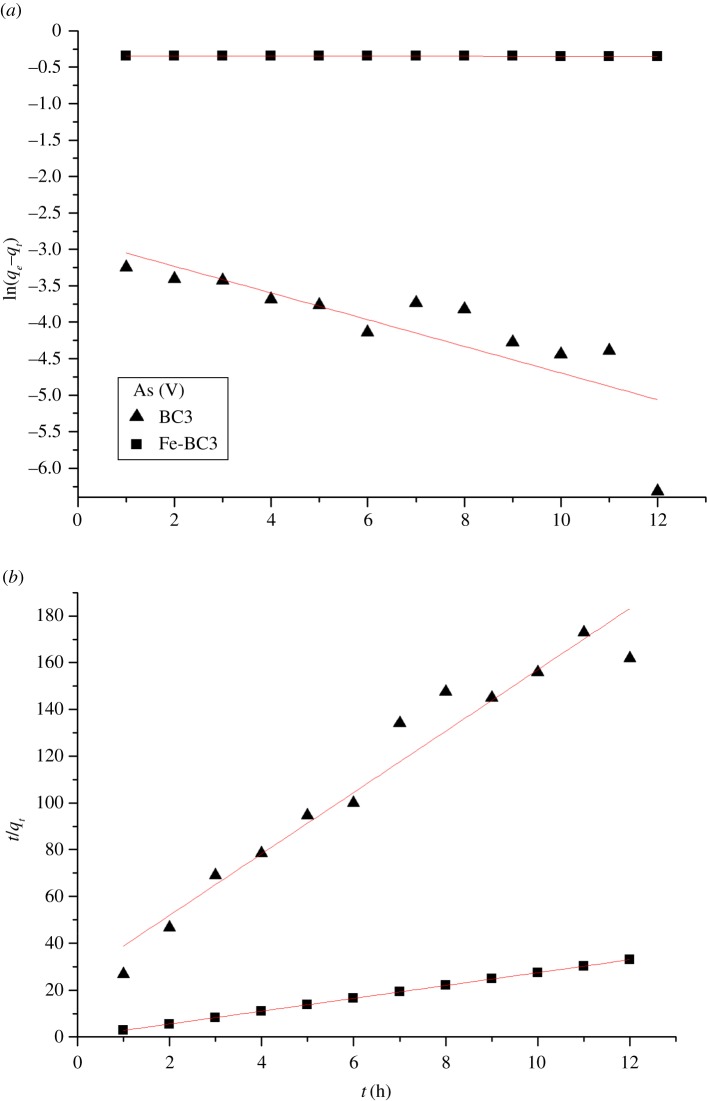
(*a*) Quasi-first-order kinetics and (*b*) Pseudo-second-order kinetics of As on: BC3 and Fe-BC3 at pH = 5.0–6.0; initial concentration = 3.668 mg l^−1^; adsorbent dosage = 0.3 g/30 ml and temperature = 25°C.

**Figure 4.**
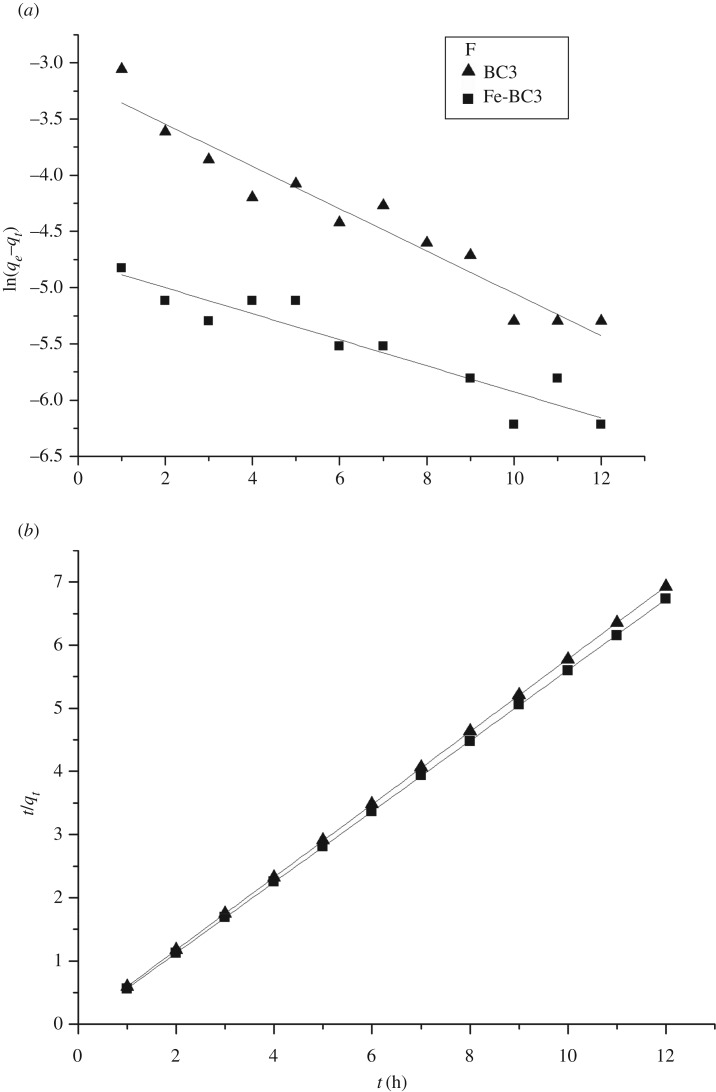
(*a*) The change of adsorption quantity of F^−^ with time, (*a*) Quasi-first-order kinetics and (*b*) Pseudo-second-order kinetics of F^−^ on: BC3 and Fe-BC3 at pH = 5.0–6.0; initial concentration = 19.0 mg l^−1^; adsorbent dosage = 0.3 g/30 ml and temperature = 25°C.

The effect of contact time was obtained using BC3 and Fe-BC3 as adsorbents at a pH of 5.0–6.0 and with an initial As(V) concentration of 3.668 mg l^−1^ and F^−^ concentration of 19.0 mg l^−1^, as shown in figures 3*a* and 4*a*. It was found that the reaction was almost saturated in the first 60 min and that the adsorption rate decreased considerably over time.

Figures 3 and 4 also show the results of fitting the experimental data to the quasi-first-order and pseudo-second-order models. The values of calculated *q_e_* (*q_e_*_.Calcd._), *k*_1_, *k*_2_ and the correlation coefficient *R*^2^ are listed in table 1. The *q_e_*_.Calcd_. determined from the quasi-first-order model is not in good agreement with the experimental values of *q_e_*_.Exp._, as shown in table 1, which indicates that the quasi-first-order model does not sufficiently represent the adsorption kinetics of As(V) and F^−^ onto biochar. By contrast, the calculated *q_e_*_.Calcd._ values from the pseudo-second-order model showed good agreement with the experimental values (*q_e_*_.Exp._). Therefore, it can be concluded that the pseudo-second-order model more adequately represents the adsorption kinetics of As(V) and F^−^ on BC3 and Fe-BC3 than the quasi-first-order model.

**Table 1.** Quasi-first-order and pseudo-second-order adsorption rate constant, calculated (*q_e_*_.Clcd._) and experimental (*q*_e.Exp*.*_) values for the adsorption of As (V) and F^−^ on BC3 and Fe-BC3 at 25°C.

			quasi-first-order			pseudo-second-order		
	biochar	*q_e.Exp._* (mg g^−1^)	*K*_1_ (h^−1^)	*q_e.Clcd_*_._ (mg g^−1^)	*R*^2^	*K*_2_ × 10^−3^ (g mg^−1^ h^−1^)	*q_e.Clcd._* (mg g^−1^)	*R*^2^
As (V)	BC3	0.0742	0.1831	0.0569	0.62509	6.7210	0.076	0.94687
	Fe-BC3	0.3648	2.965 × 10^−4^	0.7068	0.91089	20.1043	1.069	1
F^−^	BC3	1.732	0.18829	0.0421	0.92683	15.423	1.737	0.99999
	Fe-BC3	1.788	0.11579	8.489 × 10^−3^	0.8633	54.599	1.786	0.99999

#### 3.2.2. Adsorption isotherms

**Figure 5.**
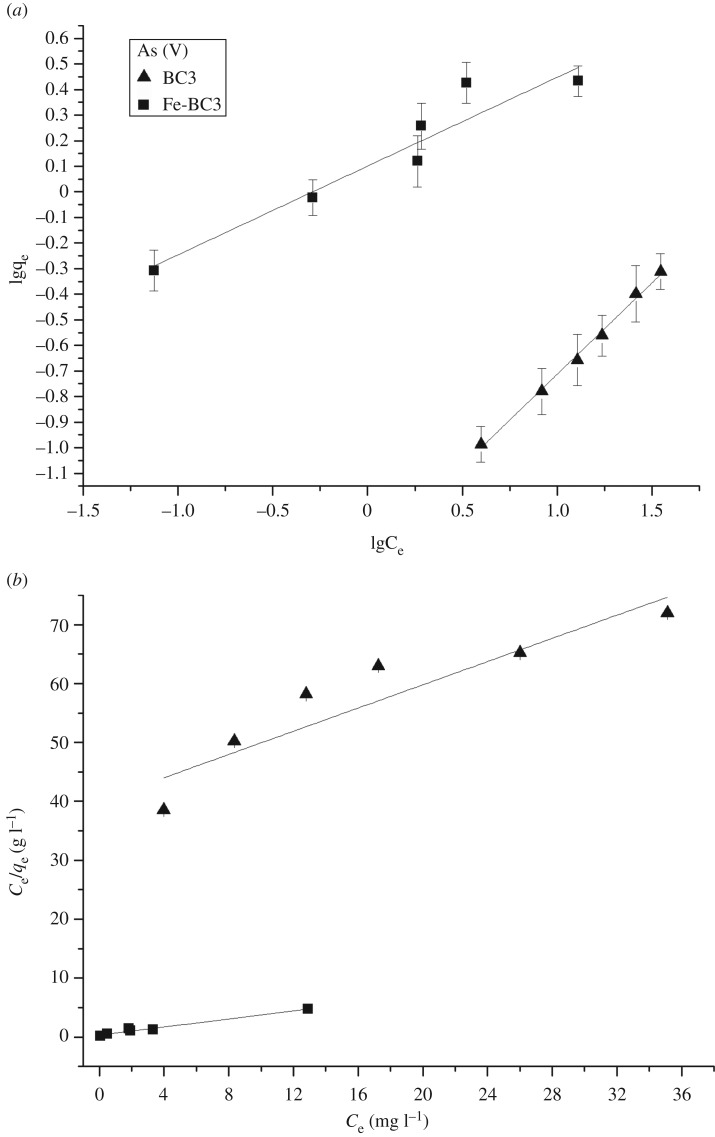
(*a*) Freundlich isotherm-model and (*b*) Langmuir isotherm-model fits for adsorption of As on BC3 and Fe-BC3 at pH = 5.0–6.0; initial concentration = 5–40 mg l^−1^; adsorbent dosage = 0.30 g/30 ml; temperature = 25°C.

**Figure 6.**
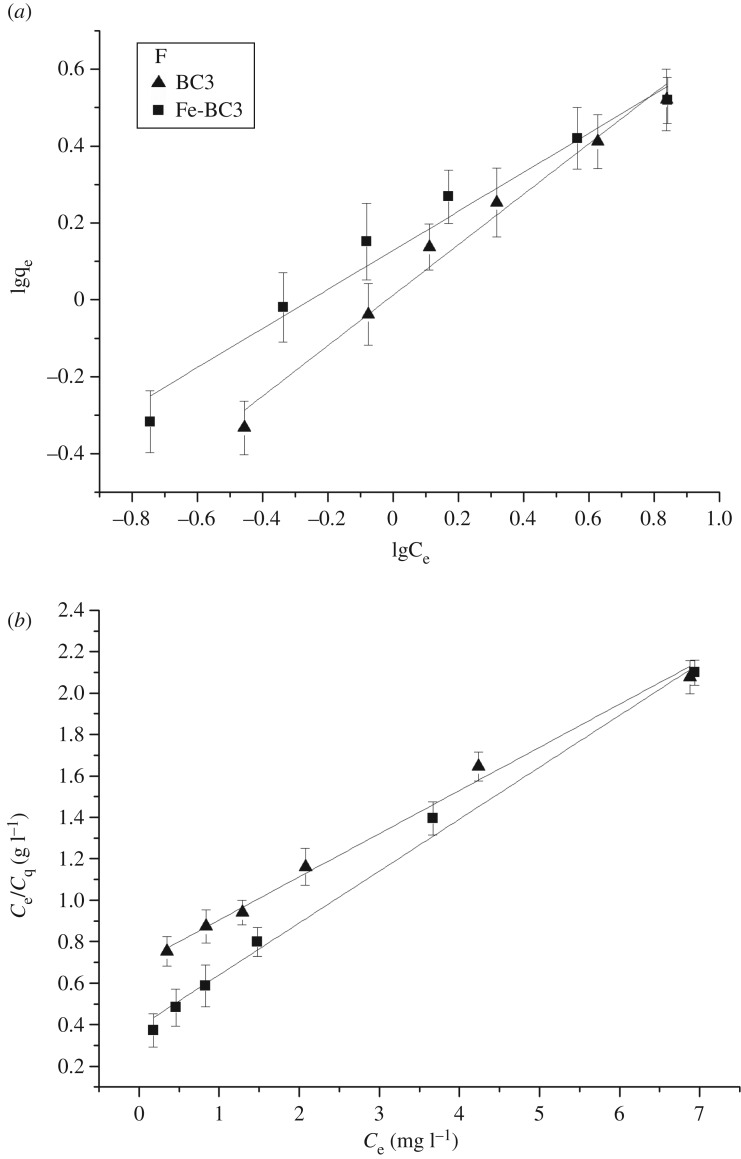
(*a*) Freundlich isotherm-model and (*b*) Langmuir isotherm-model fits for adsorption of F^−^ on BC3 and Fe-BC3 at pH = 5.0–6.0; initial concentration = 5–40 mg l^−1^; adsorbent dosage = 0.30 g/30 ml; temperature = 25°C.

Figures 5 and 6 show the adsorption isotherms of As(V) and F^−^ onto BC3 and Fe-BC3. The results of the fitted data are shown in table 2. As evident from figures 5 and 6, the adsorption capacities of BC3 and Fe-BC3 both increase with an increasing initial concentration of As(V) and F^−^. The adsorption capacity of Fe-BC3 for As(V) is significantly higher than that of BC3 at the same initial concentration. However, for F^−^, the adsorption capacity of Fe-BC3 increases slightly compared to that of BC3. Table 2 shows that the fitting coefficient *R*^2^ for the Freundlich adsorption isotherm for As (V) onto BC3 was 0.9941, which was higher than the Langmuir adsorption isotherm (*R*^2^ = 0.8213). By contrast, the fitting coefficient *R*^2^ of the Langmuir adsorption isotherm for Fe-BC3 (0.9739) was only slightly higher than that of the Freundlich adsorption isotherm (*R*^2^ = 0.9003). These results indicate that the Freundlich isotherm model is more suitable for describing the adsorption of As(V) onto BC3, while the Langmuir isotherm model is more suitable for describing the adsorption of As(V) onto Fe-BC3. The *R*^2^ of the two adsorption isotherms describing sorption of F^−^ onto BC3 and Fe-BC3 all exceed 0.96, which indicates that both isotherm models are suitable for describing the adsorption of F^−^ onto BC3 and Fe-BC3.

**Table 2.** Parameters of Langmuir and Freundlich isotherms for As (V) and F^−^ adsorption onto BC3 and Fe-BC3 at 25°C.

		values	
	model	BC3	Fe-BC3
As (V)	Freundlich		
	*K_F_* (mg g^−1^)	0.0366	1.2728
	*n* (g l^−1^)	1.3875	2.7835
	*R*^2^	0.9941	0.9003
	Langmuir		
	*q*_max_ (mg g^−1^)	1.0497	2.9257
	*K_L_* (l mg^−1^)	0.023	0.9617
	*R*^2^	0.8213	0.9739
F^−^	Freundlich		
	*K*_F_ (mg g^−1^)	1.0230	1.3633
	*n* (g l^−1^)	1.5289	1.9348
	*R*^2^	0.9801	0.9656
	Langmuir		
	*q*_max_ (mg g^−1^)	4.851	3.928
	*K_L_* (l mg^−1^)	0.2932	0.6698
	*R*^2^	0.9917	0.9924

The *q*_max_ of As(V) adsorbed onto Fe-BC3 was 2.9257 mg g^−1^, which is 2.79 times the *q*_max_ of BC3 (1.0497 mg g^−1^). This result indicates that Fe plays a pivotal role in the adsorption of As(V), which is probably because the presence of Fe increases the complexation and electrostatic interaction between As(V) and biochar [17]. The *q*_max_ of F^−^ adsorbed onto Fe-BC3 was 3.928 mg g^−1^, which is lower than the *q*_max_ of BC3 (4.851 mg g^−1^). This behaviour is probably because ion exchange is the mechanism of adsorption of fluorine by biochar [18], and the specific surface area and the total pore volume of Fe-BC3 decreased compared with BC3 (table 3).

**Table 3.** The surface area, average pore size and total pore volume of BC1, BC2, BC3 and Fe-BC3.

biochar	surface area (m^2^ g^−1^)	average pore size (nm)	total pore volume (cm^3^ g^−1^)
BC1	37.944	41.2067	0.0782
BC2	49.687	49.4962	0.12230
BC3	100.316	28.5819	0.1434
Fe-BC3	79.493	32.9245	0.1309

### 3.3. Biochar characterization

#### 3.3.1. FTIR analysis

**Figure 7.**
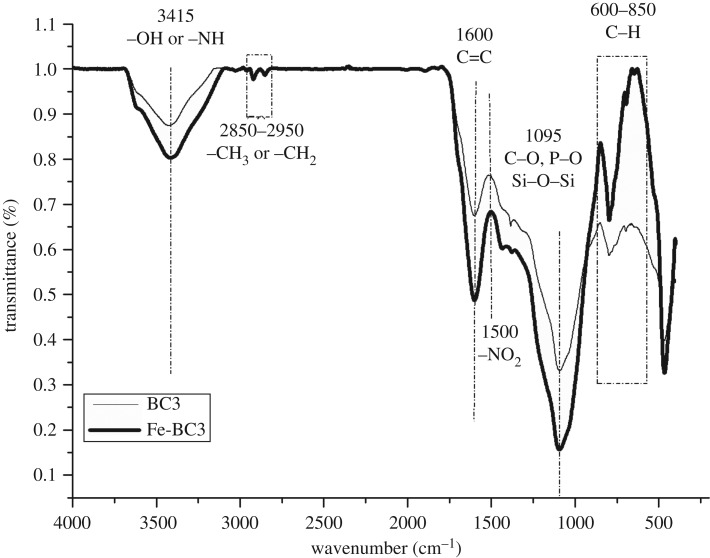
FTIR spectra of BC3 and Fe-BC3.

The FTIR spectrum of BC3 and Fe-BC3 is shown in figure 7. As shown in the figure, the peak of Fe-BC3 is more intense at the same wavenumber than that of BC3, which indicates the formation of compounds with stronger bonds for the former biochar. The broad and less intense absorption peaks at 3415 cm^−1^ are mainly due to the vibration of –NH and –OH contained in amines, amides, alcohols, phenols and so on [19]. The symmetric and antisymmetric vibrations of –CH_2_ and –CH_3_ on aliphatic hydrocarbons are mainly located between 2950 and 2850 cm^−1^. Biochars showed two unobvious absorption peaks in this region, indicating that the carbonization process had converted –CH_2_ and –CH_3_ to volatile matter or fixed carbon. The peak at 1600 cm^−1^ is predominantly attributed to the C=C skeleton in the aromatic structure [20]. Lignin is present in yak dung, and aromatic condensation of organic species can occur in biochars, so the C=C bonds exhibit vibrates in that region. The vibration of –NO_2_ is observed at 1510 cm^−1^ possibly because the biochar was washed with HNO_3_ after pyrolysis. Vibrations of Si–O–Si, a functional group of mineral components in biochars, are found and mainly occur between 1100 and 1000 cm^−1^, and some P–O and C–O vibrations exist in this region as well [21]. The absorption peak at 850–600 cm^−1^ is mainly composed of C–H vibrations aromatic rings or heterocyclic materials [22].

#### 3.3.2. XRD analysis

**Figure 8.**
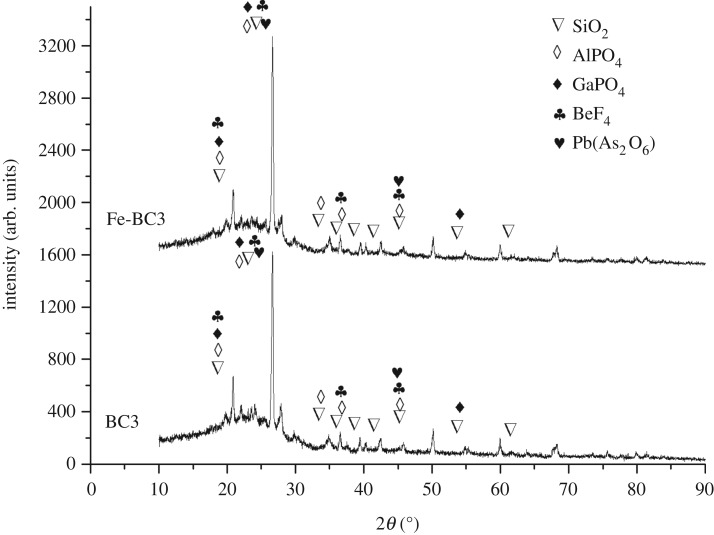
XRD diffraction pattern of BC3 and Fe-BC3.

The XRD diffraction patterns of BC3 and Fe-BC3 are shown in figure 8. The main peaks at the highest intensity of *θ* = 26.22 confirm the presence of silica (quartz). The XRD results suggested that the overall structure of BC3 and Fe-BC3 did not change significantly, as indicated by the peaks occurring at the same diffraction angle. The phase components contained in BC3 and Fe-BC3 are mainly SiO_2_, AlPO_4_, GaPO_4_ and so on. This finding is consistent with the FTIR results, which showed that the samples contained Si–O–Si and P–O functional groups.

#### 3.3.3. SEM analysis

**Figure 9.**
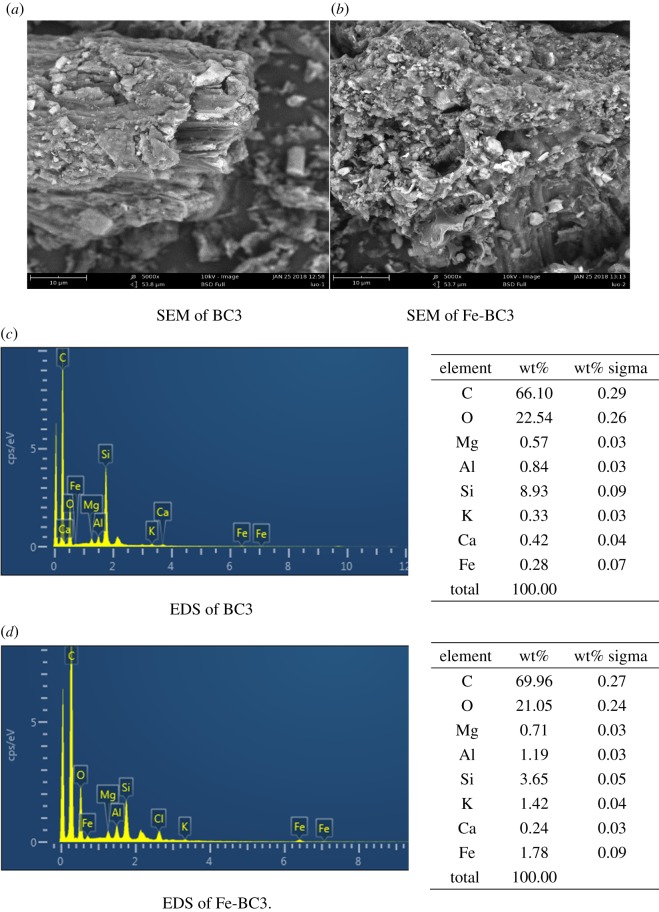
SEM micrographs and EDS of BC3 and Fe-BC3.

Figure 9 shows the scanning electron microscope images and EDS analysis for BC3 and Fe-BC3. The surface of BC3 is smoother, and the distribution of pores is more regular. There are more adsorption sites on the surface of Fe-BC3. A more porous structure can be observed on BC3 compared with Fe-BC3, which supports the values found for the surface area shown in table 3.

## 4. Conclusion and expectation

The biochars and modified biochars prepared from yak dung have a large specific surface area, which is beneficial to the adsorption of fluoride and arsenic. A quasi-second-order kinetic model can well describe the adsorption behaviour of ions on the surface of biochar. At the same time, the adsorption isotherms of fluoride and arsenic on biochar can be fitted well by Freundlich and Langmuir models, indicating that the adsorption of these elements onto biochar is a combination of homogeneity and heterogeneity.

The results show that it is feasible to remove fluoride and arsenic from geothermal water using biochar produced by pyrolysis of yak dung. Further studies are necessary to research different modification methods for biochar to increase its adsorption capacity. Further studies on the reutilization of biochar and the effects of other ions (such as Ca^2+^, Mg^2+^ and SO_4_^2−^) on adsorption are needed.

## Supplementary Material

The experimental datas

## Data accessibility

The datasets supporting this article have been uploaded as part of the supplementary material.
