# Supplementary material for: Simultaneous removal of fluoride and arsenic in geothermal water in Tibet using modified yak dung biochar as an adsorbent
Source: R Soc Open Sci. 2018 Nov 21;5(11):181266. doi: 10.1098/rsos.181266 (PMC6281945; doi:10.1098/rsos.181266)
Supplement: The experimental datas [file rsos181266supp1.docx]

1. Adsorption isotherm data of As

| C_0_(mg/L) | 5 | 10 | 15 | 20 | 30 | 40 |
| --- | --- | --- | --- | --- | --- | --- |
| C_e_(BC3) (mg/L) | 3.970 | 8.339 | 12.80 | 17.26 | 26.01 | 35.12 |
| C_e_(FeBC3)  (mg/L) | 0.0749 | 0.5158 | 1.837 | 1.926 | 3.328 | 12.91 |

1. Adsorption isotherm data of F

| C_0_(mg/L) | 5 | 10 | 15 | 20 | 30 | 40 |
| --- | --- | --- | --- | --- | --- | --- |
| C_e_(BC3) (mg/L) | 0.35 | 0.84 | 1.29 | 2.08 | 4.24 | 6.88 |
| C_e_(FeBC3)  (mg/L) | 0.18 | 0.46 | 0.83 | 1.48 | 3.67 | 6.94 |

1. Sorption kinetics data of As (C_0_=3.668mg/L)

| t(h) | C_e_(BC3) (mg/L) | C_e_(FeBC3)  (mg/L) |
| --- | --- | --- |
| 1/12 | 3.509 | 0.1911 |
| 1/6 | 3.509 | 0.1111 |
| 1/4 | 3.436 | 0.0971 |
| 1/3 | 3.411 | 0.0951 |
| 1/2 | 3.402 | 0.0640 |
| 2/3 | 3.315 | 0.0623 |
| 5/6 | 3.274 | 0.0787 |
| 1 | 3.295 | 0.0460 |
| 2 | 3.238 | 0.0414 |
| 3 | 3.233 | 0.0388 |
| 4 | 3.158 | 0.0376 |
| 5 | 3.139 | 0.0355 |
| 6 | 3.067 | 0.0335 |
| 7 | 3.146 | 0.0320 |
| 8 | 3.126 | 0.0301 |
| 9 | 3.047 | 0.0295 |
| 10 | 3.026 | 0.0229 |
| 11 | 3.032 | 0.0203 |
| 12 | 2.926 | 0.0262 |

1. Sorption kinetics data of F (C_0_=19.0mg/L)

| t(h) | C_e_(BC3) (mg/L) | C_e_(FeBC3)  (mg/L) |
| --- | --- | --- |
| 1/12 | 3.96 | 3.43 |
| 1/6 | 3.35 | 2.80 |
| 1/4 | 2.84 | 2.06 |
| 1/3 | 2.44 | 1.77 |
| 1/2 | 2.20 | 1.48 |
| 2/3 | 2.15 | 1.28 |
| 5/6 | 2.07 | 1.22 |
| 1 | 2.10 | 1.20 |
| 2 | 1.90 | 1.20 |
| 3 | 1.84 | 1.18 |
| 4 | 1.78 | 1.22 |
| 5 | 1.80 | 1.18 |
| 6 | 1.75 | 1.17 |
| 7 | 1.77 | 1.20 |
| 8 | 1.73 | 1.14 |
| 9 | 1.72 | 1.19 |
| 10 | 1.68 | 1.12 |
| 11 | 1.68 | 1.12 |
| 12 | 1.68 | 1.18 |
